# The Glycosyltransferases of LPS Core: A Review of Four Heptosyltransferase Enzymes in Context

**DOI:** 10.3390/ijms18112256

**Published:** 2017-10-27

**Authors:** Joy M. Cote, Erika A. Taylor

**Affiliations:** Department of Chemistry, Wesleyan University, Middletown, CT 06459, USA; jcote@wesleyan.edu

**Keywords:** LPS, lipopolysaccharide, heptosyltransferase, protein dynamics, glycosyltransferase, GT-B, inhibitor design

## Abstract

Bacterial antibiotic resistance is a rapidly expanding problem in the world today. Functionalization of the outer membrane of Gram-negative bacteria provides protection from extracellular antimicrobials, and serves as an innate resistance mechanism. Lipopolysaccharides (LPS) are a major cell-surface component of Gram-negative bacteria that contribute to protecting the bacterium from extracellular threats. LPS is biosynthesized by the sequential addition of sugar moieties by a number of glycosyltransferases (GTs). Heptosyltransferases catalyze the addition of multiple heptose sugars to form the core region of LPS; there are at most four heptosyltransferases found in all Gram-negative bacteria. The most studied of the four is HepI. Cells deficient in HepI display a truncated LPS on their cell surface, causing them to be more susceptible to hydrophobic antibiotics. HepI–IV are all structurally similar members of the GT-B structural family, a class of enzymes that have been found to be highly dynamic. Understanding conformational changes of heptosyltransferases are important to efficiently inhibiting them, but also contributing to the understanding of all GT-B enzymes. Finding new and smarter methods to inhibit bacterial growth is crucial, and the Heptosyltransferases may provide an important model for how to inhibit many GT-B enzymes.

## 1. Introduction

Well before the discovery of penicillin, bacteria have been evolving to resist natural antibiotics and other extracellular threats [1]; however, advances in medical techniques and over use of antibiotics has lead to an exponential increase in resistance. The resulting bacteria that are resistant to multiple antimicrobial agents are regarded as one of the biggest threats to global health, food security and development by both the World Health Organization (WHO) and Centers for Disease Control and Prevention (CDC) [2,3]. Gram-negative bacteria are of particular concern because their peptidoglycan is protected behind the bacterium’s outer membrane (OM). Furthermore, the physical properties of the OM enhance bacterial survival in diverse environments and while also limiting the uptake of many drugs [4].

The overall organization of the OM is largely conserved, despite some variability between different Gram-negative bacteria. Typically, the OM contains a phospholipid bilayer with the extracellular leaflet being composed of a mixture of lipopolysaccharides (LPS), lipoproteins, and oligosaccharides [5,6,7]. LPS are the primary component of the OM in most species of Gram-negative bacteria and have been shown to play an important role in cell motility, intestinal colonization, bacterial biofilm formation, and antibiotic resistance [8,9]. This makes understanding the role of LPS in host-pathogen interactions an area of great interest, especially in the development of therapeutic agents for the treatment of gram-negative bacterial infections [10].

**Figure 1:**
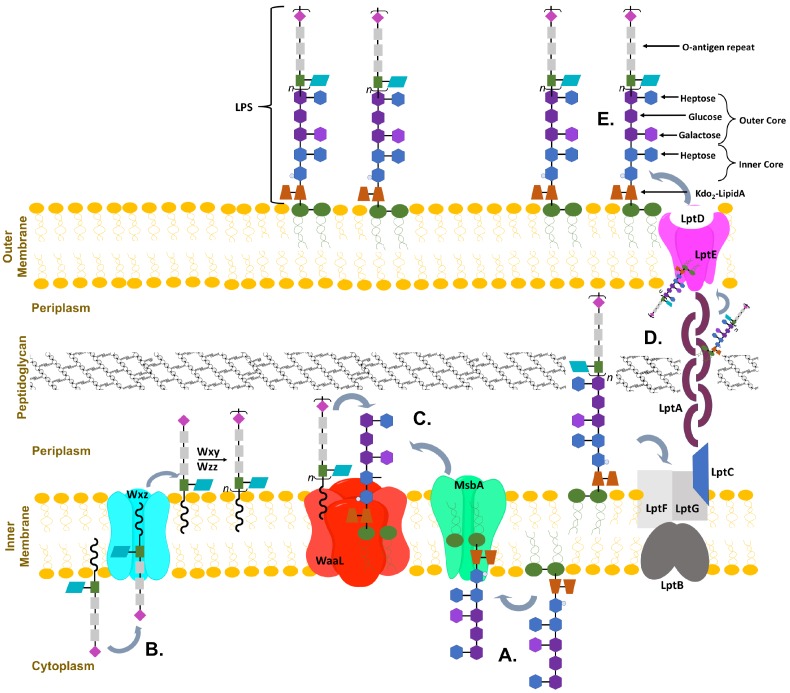
Representative organization of Gram-negative bacterium from *Escherichia coli* membrane. (**A**) demonstrates the sequential addition of inner core sugars to Kdo${}_{2}$-lipid A anchored into the inner membrane; (**B**) Represents the formation of the O-antigen repeat also formed in the inner membrane; (**C**) Once both are complete, they are flipped into the periplasm and the O-antigen repeats are attached to the top of the core; (**D**) The whole lipopolysaccharides (LPS) is then transported across the periplasm and peptidoglycan layer; (**E**) finally embedding into the outer membrane.

The LPS is composed of three main sections: a hydrophobic lipid A anchored to the membrane, a core oligosaccharide containing octulose and heptose sugar moieties, and a repeating O-antigen region containing a diversity of sugars that are unique to bacterial cell surfaces (including pentoses, deoxy-hexoses, lactyl functionalized hexoses, heptoses and nonuloses) [11,12,13]; these components vary slightly between different bacteria [9,14]. The core oligosaccharide is further divided into the inner and outer core; the inner core is highly conserved and proximal to lipid A whereas the outer core is more variable. It is possible that the evolutionarily preserved structure of the inner core may be crucial for establishing the barrier function of the OM [15]. As can been seen in a schematic of *E. coli* OM biosynthesis, the complex synthesis and transportation of LPS involves many proteins (Figure 1) [7,16,17]. Sequential glycosyl transfer from nucleotide sugar precursors by membrane associated (or proximal) glycosyltransferases (GT) on the cytoplasmic face of the plasma membrane form the inner and outer core which is then transported to the periplasm where the fully formed O-antigen repeat is attached and the full LPS is exported to the outer leaflet [7,9,17]. Mutations in the biosynthesis of LPS are often lethal to bacteria, with the minimalistic structure required for secretion of LPS to the outer membrane being Kdo${}_{2}$-lipid A (lipid A with two 3-deoxy-d-manno-octo-2-ulosonic acid (Kdo) sugar moieties attached) [9,18,19]. Truncation of the LPS by mutations to the inner core display a deep-rough phenotype and exhibit hypersensitivity to hydrophobic antibiotics and detergents [4,20,21].

## 2. Glycosyltransferases

Glycoslytransferases (GTs) are enzymes that catalyze the addition of various saccharides onto other biomolecules. GTs encompass a large group of enzymes that have similar structural scaffolds, but have evolved to utilize a vast diversity of substrates. Often, GTs act sequentially in order to build a complex polymer—the product of one GT will then be the acceptor substrate for the following GT. Many cellular functions such as: energy storage, cell wall structure, cell-cell interactions, signaling, host-pathogen, and protein glycosylation are dependent upon complex carbohydrates and polysaccharides. Due to this, biosynthesis of these chemically diverse oligosaccharides and polysaccharides require the use of multiple GTs [22,23,24,25].

### 2.1. Glycosyltransferase Structural Folds

**Figure 2:**
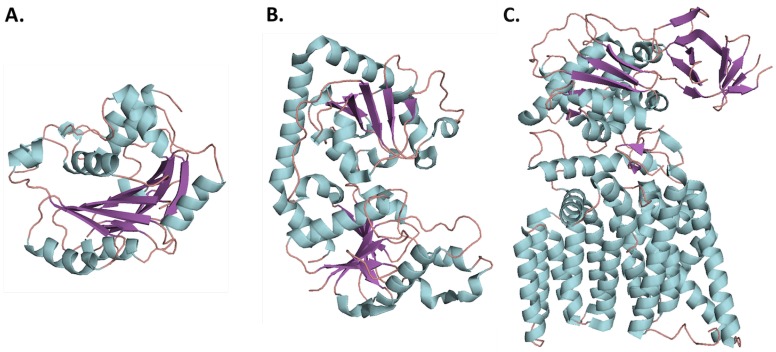
Representative folds of the first glycosyltransferase (GT) enzymes crystallized in each structural family: loops, α-helices, and β-sheets are colored salmon, cyan, and purple respectively. (**A**) GT-A fold represented by SpsA from *Bacillus subtilus*, PDB: 1QGQ; (**B**) GT-B fold represented by bacteriophage T4 β-glucosyltransferas, PDB: 1JG7; (**C**) GT-C fold represented by PglB from *Campylobacter lari*, PDB: 3RCE.

Presently, there are over 300,000 known and putative GTs according to CAZY.org (Carbohydrate-Active enZYmes Database) and the number is ever growing [26]. Although GTs have diverse sequences, they can be characterized into three structural classes: GT-A, GT-B, and GT-C (Figure 2). Despite their differences, GTs catalyze the formation of a glycosidic bond, where a high-energy sugar nucleotide donates a monosaccharide to an acceptor molecule [27]. This acceptor can be a variety of molecules, such as oligosaccharides, monosaccharides, proteins, lipids, and others [23].

#### 2.1.1. GT-A Structural Fold

SpsA from *(Bacillus subtilis*) was the first enzyme to be crystallized and characterized with a GT-A fold [28]. This structural family is characterized by two tightly packed domains, comprised of two βαβRossman-like folds, that are closely associated to form a continuous central β-sheet (Figure 2A). The close proximity of the folds lead many to describe the GT-A fold as a single domain, however there are distinct binding sites for the two substrates [25]. A short N-terminal domain binds the donor substrate and C-terminal domain is an open groove that binds an acceptor substrate.

GT-A enzymes typically contain two Asp residues separated by a non-conserved amino acid (DXD motif), that is located on a loop connecting the central β-sheet to an additional smaller β-sheet. A divalent cation interacts with one or both of the Asp residues and is essential for stabilization of the pyrophosphate group of the donor substrate. Typically absence of the cation renders the enzyme inactive, however there are a small number of GT-A enzymes where a DXD motif and cation are not required [29]. Additionally, in order for both substrates to bind and for catalysis to occur a conformational change is required. Specifically, the loops adjacent to the active site, often adopt a variety of conformations to assist in binding the substrate and performing chemistry [30].

#### 2.1.2. GT-B Structural Fold

Similar to the GT-A structural class, the GT-B protein contains two βαβRossman-like domains. Unlike GT-A proteins, in the GT-B structural class the two domains are connected by a linker region with a deep cleft containing the active site separating the two domains (Figure 2B). Donor substrate binds to the C-terminal domain, while the N-terminal domain binds the acceptor substrates. A large domain movement is required for catalysis to occur in many GT-B enzymes [25,31,32,33,34]. There are no divalent metal ions or DXD motif in GT-B enzymes, and it is believed that the pyrophosphate is stabilized by charged and polar residues as well as the natural dipole of the α-helices located in the donor substrate binding site [24].

A DNA-modifying β-glucosyltransferase was the first GT-B to be structurally characterized, and was shown to have both an open and closed conformation. The closed conformation is at least in part caused by binding of donor substrate uridine diphosphate glucose (UDP-glucose) [32]. Nonetheless, donor substrate binding does not alway induce a conformational change in GT-B enzymes. For instance, heptosyltransferase I (HepI), which has been crystallized with and without an analog to its donor substrate, ADP-l-(glycero)-d-(manno)-heptose (ADPH), shows no donor substrate induced closure [31]. Some GT-B enzymes are closed with acceptor bound thus, the order or ligand state required for closure does not seem to be universal across the family.

#### 2.1.3. GT-C Structural Fold

Until recently, the third structural fold (GT-C) was only predicted on the bases of sequence analysis [35]. In 2011, the first GT-C structure was published for a bacterial oligosaccharyltransferase from *Campylobacter lari*, comprised of 2 domains: a 13 transmembrane domain and a periplasmic domain containing a mix of α/β folds [36]. Both GT-A and GT-C enzymes have a DXD motif, however the location of the DXD tripeptide in GT-C family is at the carboxy-terminal end of the first transmembrane helix. A small patch of hydrophobic amino acids following the helix is common. Although this arrangement is similar to that of the DXD signiture in GT-A structural fold, there is no conservation of sequence between these two regions [24,36].

#### 2.1.4. Catalytic Mechanisms

**Figure 3:**
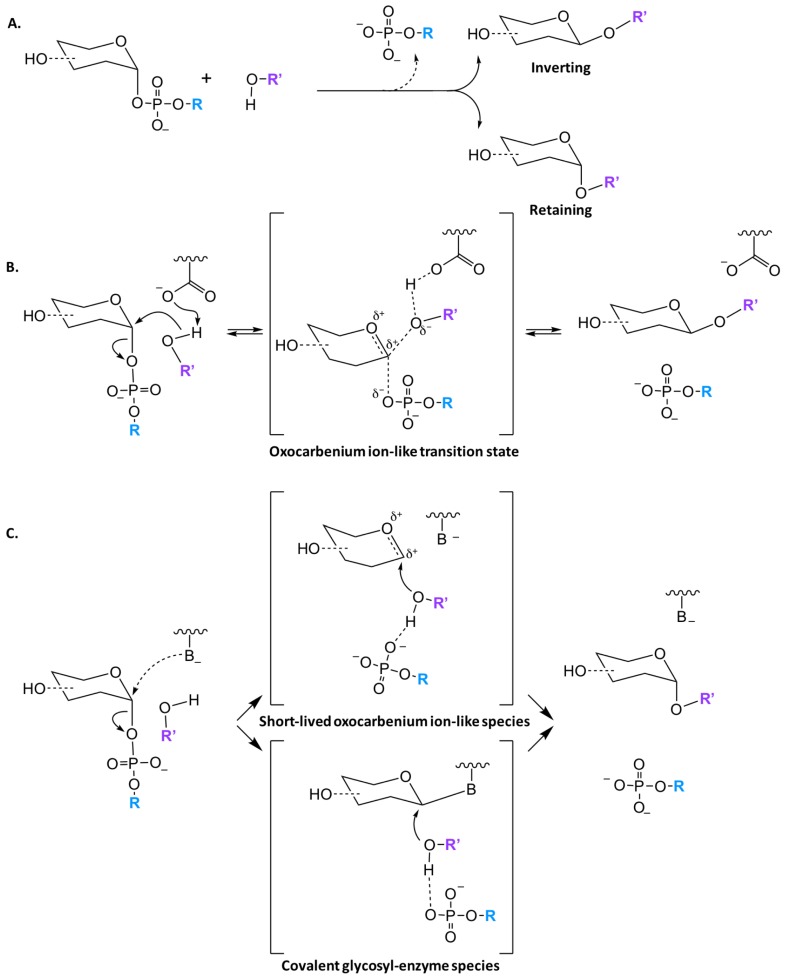
Proposed catalytic mechanism of GT enzymes. (**A**) The transfer of a sugar moiety is performed with either inversion or retention of the anomeric carbon in respect to the sugar donor substrate; (**B**) Schematic of S_N_1-like mechanism for inverting GTs, where a single oxocarbenium ion-like transition state is formed; (**C**) There are currently two mechanisms for retaining GTs enzymes either through the formation of a short-lived oxocarbenium ion-like species or a covalent glycosyl-enzyme species.

Regardless of the structural fold, glycosyltransferases catalyze the transfer of a glycosyl group with either inversion or retention of the stereoconfiguration at the anomeric carbon. Both GT-A and GT-B families have been found to have inverting and retaining enzymes, however all GT-C enzymes are predicted to utilize an inverting mechanism. While literature often states that inverting GTs follow a S_N_2-like mechanism, implying an uncharged transition state, it is generally accepted that the reaction has an oxocarbenium ion like transition state which is more correctly defined as a partially associated S_N_1-like mechanism (Figure 3B) [37,38]. Unlike inverting GTs, there are multiple mechanism for retaining enzymes. Initially, it was thought that all retaining enzymes proceed via a double displacement mechanism with formation of a covalent glycosyl-enzyme intermediate (Figure 3C); nevertheless, only a small percentage of GTs contain a putative nucleophilic residue that is properly located in the active site to facilitate such a mechanism [25,39,40]. There is little direct evidence for a double displacement mechanism in the literature, however Soya et. al. was able to observe glycosyl-enzyme intermediates by mass spectrometry [41]. Further experimental and computational work has shown that a front face or S_N_i (substitution nucleophilic internal-like) mechanism is likely the primary pathway utilized [25,40,42,43]. While there is more support for an S_N_i mechanism, it is generally accepted that there are two classes of retaining GTs, that are classified based on the presence or absence of a nucleophile in the active site.

## 3. Core Heptosyltransferase Enzymes

**Figure 4:**
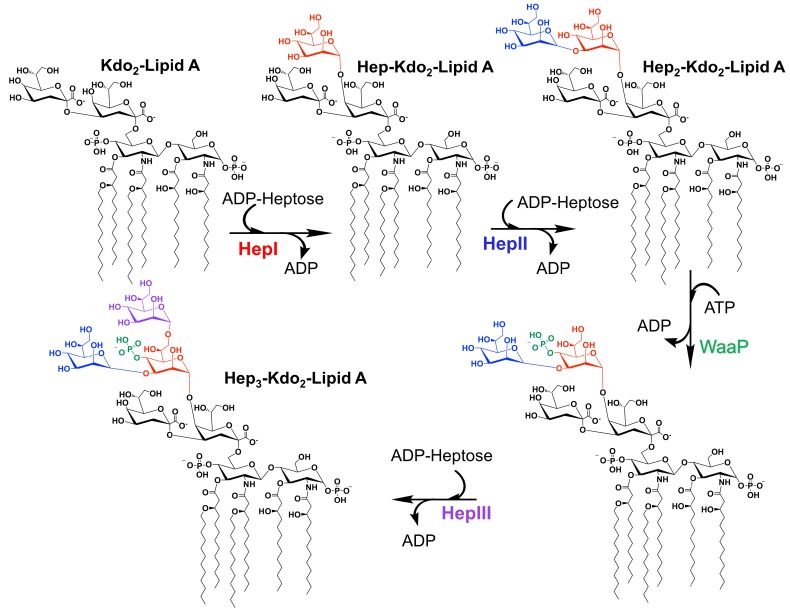
The sequential addition of three heptose moieties from ADPH catalyzed by Heptosytransferase I, II, and III (HepI, II, and III, respectively). Each moiety is color coded to match the enzyme that catalyzed the addition (red, blue, purple for HepI, II, and III, respectively). Prior to addition of the third heptose, WaaP phosphorylates the first heptose [46].

Many GTs have been extensively studied due to their biological and medical importance. Notable of these enzymes are the heptosyltransferases that are involved in the biosythesis of the LPS inner core (and in some bacteria outer core). Heptosytransferases catalyze the sequential addition of heptose moieties onto Kdo${}_{2}$-lipid A (Figure 4) and are characterized as GT-B enzymes inverting reaction mechanism [44]. As mentioned earlier, in all Gram-negative bacteria, LPS is one of the major extracellular polymeric substances protecting the cell (a schematic of which is shown in Figure 1). For many bacteria the overall structure of LPS is highly conserved. However, as one moves away from the membrane the structure variability between bacterial species increases. Thus, the inner core of LPS has low variability where the outer core varies more between bacteria. Additionally, it has been shown that the less conserved regions are not required for bacterial viability [45]. In fact, the minimal structure required for bacterial survival is Kdo${}_{2}$-lipid A - the acceptor substrate for HepI [9,18,19]. Truncation of the LPS increases the bacteria sensitivity to hydrophobic antibiotics and detergents, making the heptosyltransferases, especially HepI, novel drug targets [4,20,21].

### 3.1. Multiple Sequence Alignment (MSA) of Heptosyltransferase Enzymes

**Figure 5:**
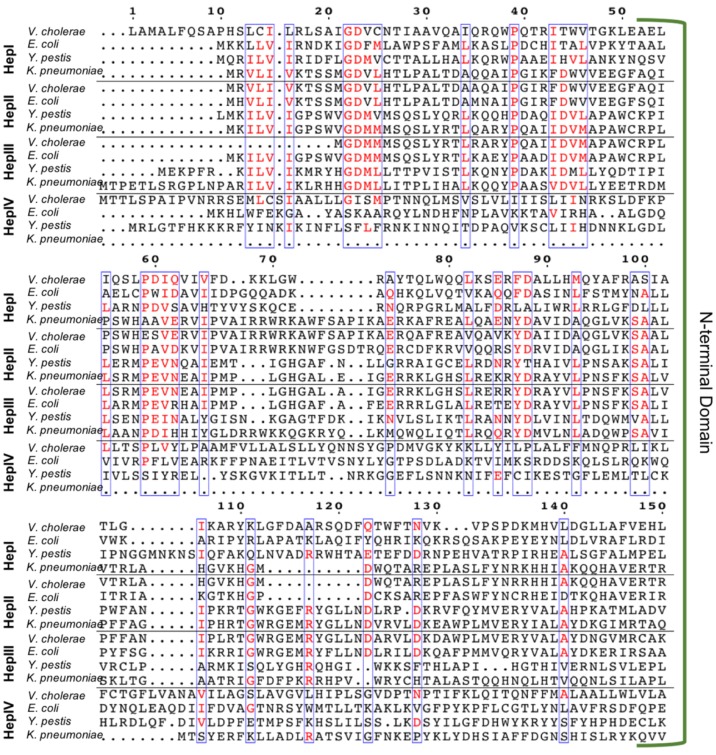

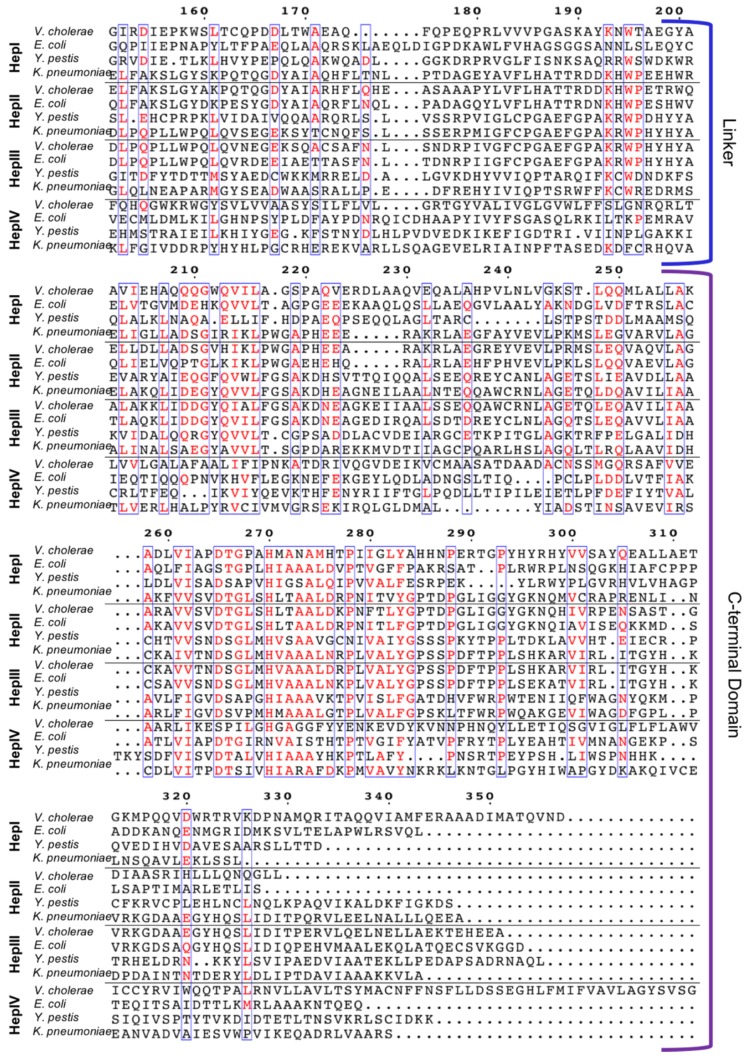
Muliple sequence alignment of HepI–HepIV from *Vibrio cholerae*, *Escherichia coli*, *Yersinia pestis*, and *Klebsiella pneumoniae* (domains for HepI *E. coli* HepI are annotated), using ClustalW 2.0 (https://www.ebi.ac.uk/Tools/msa/clustalw2/) alignment program and Espript 3.0 (ESPript—http://espript.ibcp.fr) [49,50].

Gram-negative bacteria have up to four heptosyltransferases; HepI and HepII are always present and catalyze the addition of the first two sugars of the inner core, whereas HepIII and HepIV are found only in some species (Figure 4). As a result HepI and HepII have been studied in many systems. To date, there has been little work on HepIII and even less on HepIV, despite both having been identified or suggested in *Vibrio cholerae*, *Escherichia coli*, *Yersinia pestis*, and *Klebsiella pneumoniae*. HepIII adds the third heptose to the inner core and HepIV adds a heptose moiety onto a glucose or galactose located within the outer core (Figure 1) [47,48]. A multiple seuqence alignment (MSA) of HepI–IV from *Vibrio cholerae*, *Escherichia coli*, *Yersinia pestis*, and *Klebsiella pneumoniae* shows the variability of sequence conservation among the heptosyltransferases (Figure 5). The average similarity for all 16 heptosyltransferases is about 30%, which is consistent to the percent similarity for the HepI–IV enzymes from the same organism. By comparing each homolog to *E. coli*, it can be concluded that HepI and HepII are highly conserved with percent similarities as high as 86%. HepIII and HepIV homologues have less then 46% similarity. It is perhaps unsurprising that each of the heptosyltransferases have the highest sequence similarity to their homologs rather than to the paralogs within an organism. HepIV are more divergent than HepI, most likely because the core region of LPS only varies only slightly, thus a HepI enzyme from *E. coli* and *V. cholerae* will bind more similar acceptor substrates than the corresponding HepIV enzymes [45].

**Figure 6:**
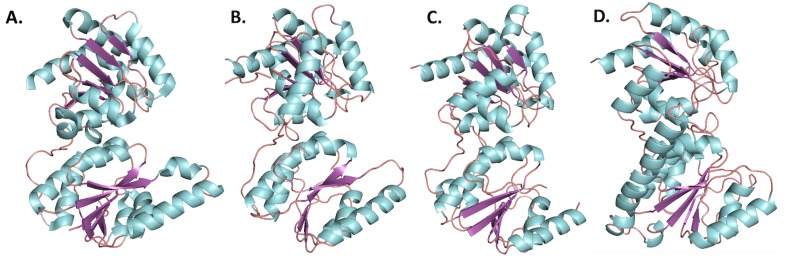
Structures of Heptosyltransferases (loops, α-helices, and β-sheets are colored salmon, cyan, and purple, respectively). (**A**) HepI from *E. coli*, PDB: 2H1H; (**B**) HepII from *E. coli*, PDB: 1PSW; (**C**) Computational model of HepIII from *E. coli* [51]; (**D**) Computational model of HepIV from *E. coli*.

Although HepI–IV are variable in their sequence, the C-terminus has the most conservation followed by the N-terminus, while the linker is highly variable, and the overall structure of heptosyltrasferases are homologous (Figure 6). In *E. coli* HepI and HepII have been crystallized, and the structure of HepIII has been computationally predicted (Figure 6A–C) [51]. A computational model of *E. coli* HepIV was created using the I-Tasser protein structure prediction program (the resulting structure is shown in Figure 6D) and it appears similar to HepI and HepII crystal structure [52,53,54]. All are GT-B proteins with the the typical βαβRossman-like domains attached by a linker. The C-terminal domain for all, binds ADPH (the donor substrate) and look nearly identical, whereas the N-terminal domain varies slightly, likely due to their variation in acceptor substrates [31,51].

**Figure 7:**
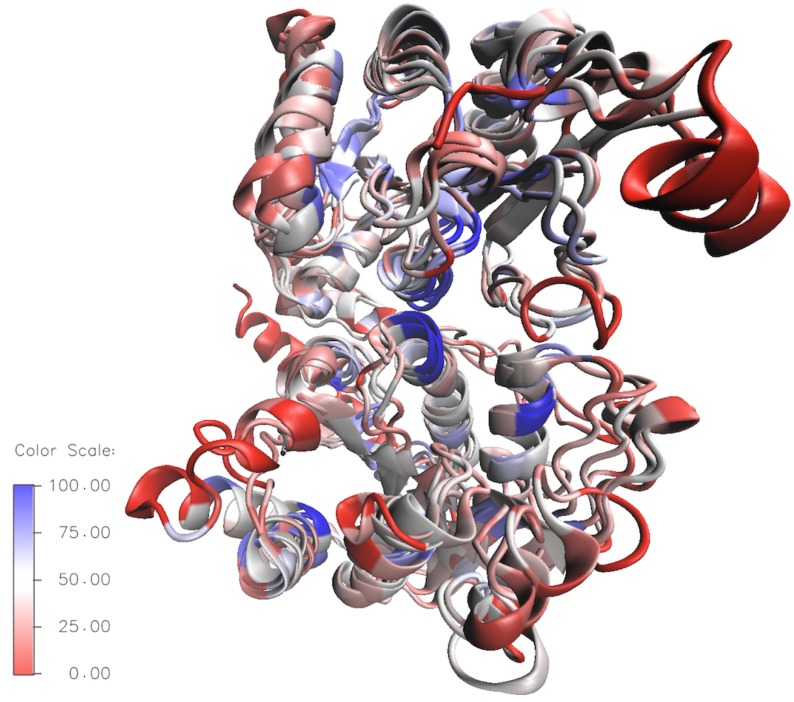
Overlay of Structurally aligned HepI–IV; residues are colored by sequence similarity [highly conserved (blue) non-conserved (red)].

To better compare the the structural variety of *E. coli* HepI–IV a sequence-based structural superposition was generated using HepI (PDB:2H1H) as the reference structure, with the VMD multiseq program. An overlay showing the conserved residues in the HepI–IV structures are displayed in Figure 7 (the blue areas indicate highly conserved regions and the red depicts non-conserved regions). By looking at the global conservation of heptosyltransferases, the interior is more conserved, while the surface residues are highly variable. Additionally, it is evident that the C-terminus (binding domain of ADPH for all heptosyltransferase enzymes) is more conserved than the N-terminal domain. The proposed catalytic base D13 is present in all the heptosyltransferases, suggesting that the mechanism of action for all heptosyltransferases are similiar [31]. Other specific residues, like K192 and D261 (which were shown by mutagenesis studies in HepI to be important for chemistry), are completely conserved for all *E. coli* heptosyltransferases as well as all *Vibrio cholerae*, *Escherichia coli*, *Yersinia pestis*, and *Klebsiella pneumoniae*. It is clear that all of the heptosyltransferase enzymes are structurally similar and many important residues are conserved not only between *E. coli* heptosyltransferase enzymes, but also in multiple bacterial species. Bacterial evolution to differentiate the LPS structure enhancing survival in different niches likely governs the sequence variability of heptosyltransferases.

### 3.2. Heptosyltransferase I

**Figure 8:**
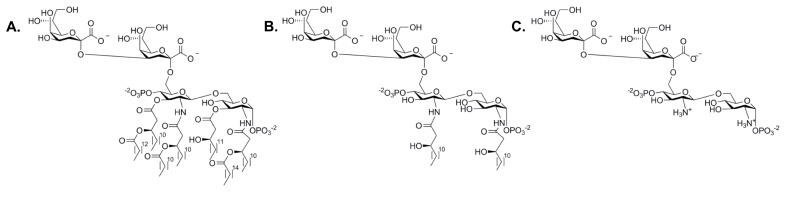
Strucutres of Kdo${}_{2}$-lipid A and analogues: (**A**) *E. coli* Kdo${}_{2}$-lipid A; (**B**) O-deacylated *E. coli* Kdo${}_{2}$-lipid A (ODLA) and (**C**) fully deacylated *E. coli* Kdo${}_{2}$-lipid A (FDLA).

*E. coli* HepI, the most characterized heptosyltransferase, can reveal insights about the function of the other heptosyltransferase enzymes. The acceptor substrate of HepI, Kdo${}_{2}$-lipid A, is the minimalistic structure required for LPS to be transported to the outer membrane. Mutations to *waaC* (*rfaC*), the gene that codes for HepI, leads to a rough phenotype LPS and an increase in sensitivity to hydrophobic antibiotics including: erthromycin, ampicillin, and novobiocin [10]. Early work on HepI sought to use alternative donor substrates as ADPH was not commercially available. ADP-mannose, GDP-manose, ADP-glucose, UDP-glucose, and UDP-galactose were tested for HepI transferase activity, only ADP-mannose was a viable alternative substrate. ADP-mannose was characterized by Kadrmas et al. to have an apparent *V*_max_ of 3 μmol/min/mg and a *K*_M_ of 1.47 mM. Kdo-lipid A (an analogue of Kdo${}_{2}$-lipid A with only one Kdo) was a poor mannose acceptor substrate; this was unexpected since the second Kdo moiety was not expected to influence activity since the first Kdo is the one being modified by HepI [55]. Later work using the native substrate ADPH showed that Kdo-lipid A was in fact a competent acceptor substrate with a *K*_M_ of 46 μM [56]. Perhaps using two alternative substrates was the reason for the poor transferase activity, and in fact Kdo-lipid A may be sufficient for the continual formation of the inner core. Interestingly, the fatty acid chains were shown to not be important for catalysis, as has been demonstrated to be necessary in other LPS biosynthetic enzymes. The substrate analogue, Kdo${}_{2}$-lipid IV_A_, although missing three fatty acid chains normally present in Kdo${}_{2}$-lipid A, gives a *K*_M_ of 4.7 μM demonstrating that the removal of fatty acid chains does not impair chemistry. Furthermore, HepI has activity with the fully deacylated and O-deacylated Kdo${}_{2}$-lipid A (ODLA and FDLA respectively, Figure 8). Both were shown to be competent substrates; native substrate had a *K*_M_ of 29 μM where the analogues displayed a *K*_M_ of 1 μM (ODLA) and 0.3 μM (FDLA) [56]. Retrospectively, it is unsurprising that the fatty acid chains would be unimportant for catalytic efficiency since they are embedded into the inner membrane in vivo and therefore should not be accessible to influence binding. Taken together with the slightly better catalytic efficiency for deacylated Kdo${}_{2}$-lipid A analogues, these observations suggest that the tetrasaccharide portion of the substrate provides HepI with the primary binding interactions required for acceptor substrate recognition.

#### 3.2.1. Crystal Structures of HepI

As mentioned earlier, the structure of HepI has been previously determined. Three different structures are available in the PDB: 2GT1, 2H1H, and 2H1F corresponding to the Apo protein, HepI·ADP-2-deoxy-2-fluoro-heptose (ADPF) complex and HepI·ADP complex, respectively [31]. By comparing the three structures it seems that HepI does not undergo a domain rotation upon donor substrate binding like other GT-B’s. However, as there is no crystal structure in complex with Kdo${}_{2}$-lipid A or any of its analogues, and it is, therefore, possible that HepI closure is induced by the acceptor substrate or by formation of the ternary complex, as has been shown by other GT-B’s. ADPF is a non-cleavable analogue to ADPH with a fluorine replacing a hydroxyl group in the 2${}^{\prime }$-position. ADPF has been shown to be an inhibitor of HepI with an IC_50_ of 30 μM [31]. Attempts to crystallize HepI with ADPH lead to co-crystallization with ADP, suggesting that HepI is capable of hydrolysis in the absence of an acceptor, a phenomenon that was observed with other glycoslytransferases [34,57]. Upon crystallization of HepI, Grizot et al. performed site-directed mutagenesis to test the importance of numerous residues on catalysis and binding of ADPH. D13A and D261A exhibit a 4688-fold and 2027-fold drop in specific activity, respectively and were suggested to be catalytic residues. K192A had a 926-fold reduction in activity and due to its location proximal to the anomeric carbon of ADPH which is where the deprotonated hydroxyl of Kdo${}_{2}$-lipid A attacks, and therefore may play an important role in binding or catalysis and binding [31].

#### 3.2.2. Inhibition of HepI

**Figure 9:**
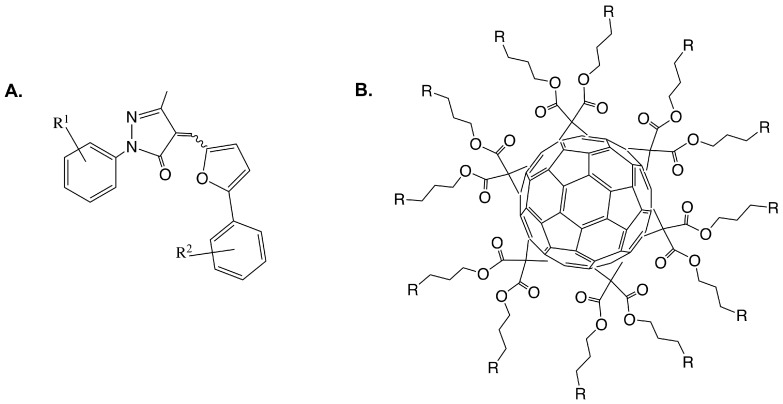
(**A**) Representative core structure of 2-aryl-5-methyl-4-(5-aryl-furan-2-yl-methylene)-2,4-dihydro- pyrazol-3-one analogues [58]; (**B**) Glycofullerene derivatives schematic [59].

One goal of understanding the heptosyltransferases is to learn how to effectively inhibit them, to date some work has been done to design inhibitors for HepI. Most inhibitors of GTs bind typically with low μM affinities, similar to *K*_M_ values of substrate. A structure-activity relationship (SAR) study was done by Moreau et. al. on a series of 2-aryl-5-methyl-4-(5-aryl-furan-2-yl-methylene)- 2,4-dihydro-pyrazol-3-one analogues (Figure 9A) [58]. In this work, computational docking and biochemical assays were used to assess binding. All compounds bound with low μM IC_50_’s and appeared to be preferentially bound close to acceptor site of Kdo${}_{2}$-lipid A, specifically near where Kdo should bind. Residues R120, H139, A140, R143, and I287 were identified as important for inhibitor binding in this analysis, suggesting the potential importance of these residues for Kdo${}_{2}$-lipid A binding. Further studies would need to be done however to test this hypothesis.

Additionally, Durka et al. published a library of synthesized multivalent glycosylated fullerene monomers and “balls” much larger than the proposed molecules previously discussed. Like the previous series, these compounds had inhibition constants in the low μM ranging from 7–47 μM, again on par *K*_M_ of substrates [59]. In 2016, the group published a second series of glycofullerenes slightly varying the fullerenes in an attempt to increase inhibition. The new fullerene compounds were competitive against Kdo${}_{2}$-lipid A and uncompetitive towards ADPH, which was unexpected because the compounds were designed to mimic ADPH heptose moiety; similar to previously discussed work, the compounds showed low μM *K_i_*. From these findings, glycoclusters mimicking Kdo with C_60_ and other multivalent scaffolds were synthesized and IC_50_ were calculated. Interestingly, high nanomolar inhibition was observed for Kdo fullerenes attached to C_60_ scaffolds, a degree of inhibition never achieved for HepI and rarely for GTs [60].

#### 3.2.3. Investigations of HepI Protein Dynamics

As mentioned earlier, a few GT-B glycosyltransferases have been shown to inter-convert between an “open” and “closed” structure [32,33,34]. Without a crystal structure of the ternary complex of HepI, it is unclear how/if HepI undergoes such an event. By looking at the crystal structure however, its is clear that the catalytic base (D13) is over 8 Å away from the anomeric carbon of ADPH which is too far away for efficient nucleophilic attack by deprotonated Kdo${}_{2}$-lipid A [31]. Thus, it was hypothesized that HepI also undergoes a conformational change during the reaction. To assess protein dynamics, HepI steady state activity was tested in a variety of viscous buffers, specifically glycerol, ethylene glycol, and PEG 8000. Microviscogens; glycerol, and ethylene glycol, both had a strong impact on *k*_cat_ which could be explained by water reorganization being required for catalysis; this suggests that HepI conformational dynamics are partially rate-limiting [61].

**Figure 10:**
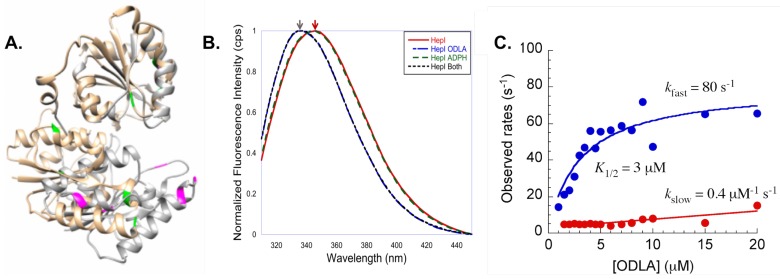
(**A**) HepI open structure (tan) and trypophan residues colored in green is superimposed with a structural model of closed HepI (gray) with tryptophans colored magenta; (**B**) Steady state intrinsic tryptophan emission spectra of HepI with and without substrates bound, blue shift is observed upon ODLA binding; (**C**) Pre-steady state kinetics of WT HepI titrated with ODLA (fast phase, blue; slow phase, red).

Additionally, intrinsic tryptophan (Trp) fluorescence spectroscopy was employed to see if substrate binding induced changes in the protein fluorescence spectra [62,63,64,65,66,67,68,69,70]. HepI has 8 tryptophan residues, and by examination of a computational model of the closed structure of HepI, many of the Trp residues appear to become more buried in the protein, suggesting that upon substrate binding there may be a change in fluorescence spectrum (Figure 10A). Fluorescence spectra were obtained with and without substrates(Figure 10B). Consistent with crystal structures, no change was observed upon ADPH binding alone. ODLA binding resulted in a 6 nm blue shift, suggesting that ODLA binding induces conformational changes that lead to one or more the the Trp residues to becoming more buried in the protein [61].

When stopped flow was used to monitor the kinetics of conformational changes in HepI, a concentration of ODLA dependent biphasic pre-steady state kinetics was observed. Two rates were observed, a fast rate which exhibits a hyperbolic dependence on ODLA concentration (saturates at 80 s^−1^), and a concentration independent slow rate of ~5 s^−1^. A two step binding mechanism of ODLA was suggested (initial collision complex between of HepI and ODLA followed one or more conformational change(s) to form the HepI·ODLA complex). Additionally, a catalytically impaired mutant (D13A) of HepI was tested and yielded the same pre-steady state kinetics. This suggests that the ODLA induced change in HepI must occur prior to chemistry [61]. Subsequently, work investigated which Trp residue(s) play(s) a role in the observed blue shift so as to better understand conformational change(s) that occur [71]. In this work, most of the eight Trp residues were mutated to phenylalanine (Phe). W62F and W116F both of which are located on the N-terminal ODLA binding domain, exhibited a reduced blue shift upon ODLA binding as compared to wild-type HepI. Additionally, these residues are located on dynamic loops (N-3 and N-7) suggesting that these loops may undergo conformational changes when ODLA binds, leading to a change in local environment of W62 and W116. Interestingly, the W217F mutant (Trp located on the C-terminal domain far from the ODLA binding site) resulted in a complete loss of the blue shift upon substrate binding. Although more experiments are need to fully understand the role of W217, upon ODLA binding, ADPH binding may be altered (ADPH is directly moved to impact W217 conformation), suggesting communication between the two domains.

**Figure 11:**
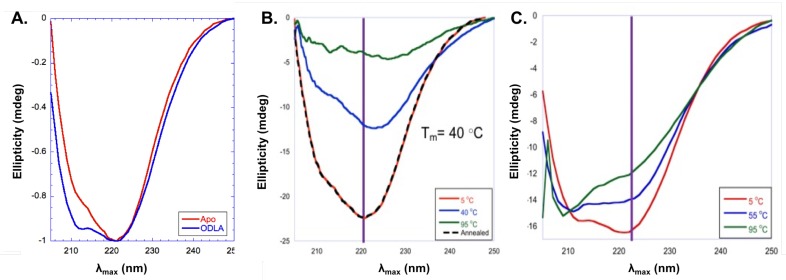
(**A**) Far-UV circular dichroism (CD) spectra of apo HepI (red), HepI with 100 μM ADPH (dark green), and HepI with 100 μM ODLA (blue) at 5 ${}^{\circ }$C. Far-UV CD Melt spectra of (**B**) apo HepI at 5 ${}^{\circ }$C (red), 40 ${}^{\circ }$C (blue), 95 ${}^{\circ }$C (green) and annealed at 5 ${}^{\circ }$C (black) and (**C**) HepI with 100 μM ODLA 5 ${}^{\circ }$C (red), 40 ${}^{\circ }$C (blue) and 95 ${}^{\circ }$C (green). Purple lines demonstate changes in ellipticity at 222 nm.

In addition to fluorescence spectroscopy, circular dichroism (CD) experiments were used to investigate structural changes of HepI [72,73]. CD spectra for HepI demonstrate a characteristic spectra for a protein with primarily αcontent with a double minimum with peaks at 222 nm and 211 nm. Interestingly, upon binding of ODLA there is an increase in the intensity of the second minimum at around 211 nm consistent with a 12% increases in α-helicity (Figure 11A). The location of these conformational changes are unknown, but most likely this is the result of structural changes of disordered loops in HepI [71].

Protein stability was also explored by CD melts experiments (taking CD spectra at varying temperatures) and monitoring unfolding of protein. As can be seen in Figure 11B,C, apo HepI has a T_M_ of 40 ${}^{\circ }$C, however upon ODLA binding there is a large increase in stability so that even at 95 ${}^{\circ }$C the protein is still mostly folded [71]. It was concluded that the formation of HepI·ODLA complex must lead to formation of hydrogen bonds and/or salt bridges (ionic interactions) between ODLA and HepI. Without cyrstalographic evidence showing where ODLA binds, its hard to determine which interactions induce such a stabilization. Examination of the HepI structure reveals that there are many positively charged residues located on dynamic loops of the N-terminal domain (where ODLA binds) which could coordinate with negatively charged phosphate and carboxylate moieties of ODLA. High salt reverses some of the HepI·ODLA complex stabilization, suggesting that ionic interactions are essential for HepI·ODLA complex stabilization [71]. In sum, these data strongly support the hypothesis of heptosyltransferases undergoing open to closed transitions.

## 4. Conclusions

With the growing need for new antibiotics to treat antibiotic resistant (and multi-resistant) bacteria, it is essential for scientists to find new and smarter ways to inhibit bacterial growth. Heptosyltransferases are important for LPS biosynthesis and the resulting resistance. Structurally HepI–IV are very similar and all adopt a GT-B structural fold. HepI can be used as a model for other heptosyltransferases and GT-B enzymes to inform inhibition strategies. Perhaps disruption of GT-B dynamics with small molecules would be an effective new strategy for inhibitor development. Additionally, dynamics disruption could potentially allow for inhibition of multiple targets which undergo similar dynamical changes (with a single drug targeting multiple enzymes). Promising work has been done to understand the function of HepI and aid in designing such an inhibitor. Ultimately, heptosyltransferases provide useful information about the GT-B structural fold and provide a model for novel methods to inhibit many GT-B enzymes.
